# Epidemiology of Severe Mental Disorders in Older Adults: A Population-Based Longitudinal Study in Aragón, Spain

**DOI:** 10.3390/healthcare14172687

**Published:** 2026-08-24

**Authors:** Alicia Muñoz-Segovia, Irene Gómez-Latorre, Iris Martín-Peña, Clara Vivó-García, Laura Lafarga-Molina, Josep-Oriol Casanovas-Marsal

**Affiliations:** 1 Short-Stay Hospitalization Unit, University Hospital of Cuenca, 16003 Cuenca, Spain; alicia95cuenca@gmail.com; 2 Short-Stay Unit, Miguel Servet University Hospital, Pº Isabel La Católica, 1-3, 50009 Zaragoza, Spain; igomezla@salud.aragon.es; 3 Sagasta-Ruiseñores Mental Health Unit, Zaragoza II Health Sector, Aragón Health Service, Pº Sagasta 52, 50006 Zaragoza, Spain; imartinp@salud.aragon.es; 4 Adult Mental Health Day Hospital, La Plana Health Department, 12002 Castellón, Spain; claravg9734@gmail.com; 5 Aragón Health Research Institute, Avda. San Juan Bosco 13, 50009 Zaragoza, Spain; jocasanovas@iisaragon.es

**Keywords:** mental disorders, incidence, health services accessibility, aged, epidemiology

## Abstract

**Highlights:**

**What are the main findings?**

- Adults aged ≥65 years showed an average annual incidence of mental disorders requiring hospital admission comparable to that of the overall adult population, but exhibited a distinct clinical profile characterised by a higher proportion of mood and organic mental disorders, disability, and polypharmacy.
- Geographical inequalities varied by age, with excess incidence among older adults concentrated in a small number of mountainous counties, suggesting that territorial context may contribute to mental health disparities.

**What are the implications of the main findings?**

- Mental health strategies should be adapted to the specific needs of older adults by integrating prevention, early detection, comprehensive geriatric assessment, and multidisciplinary care.
- Regional planning should address territorial inequalities by strengthening community mental health services and improving access to care in geographically dispersed and ageing areas.

**Abstract:**

**Background/Objectives:** Mental disorders represent a major public health challenge, particularly in ageing populations. This study aimed to analyse the incidence of mental disorders requiring hospital admission in Aragón (Spain), compare the epidemiological and clinical characteristics of adults aged ≥65 years with the overall adult population, and evaluate temporal and geographical variations in incidence. **Methods:** A retrospective population-based study included all incident cases of mental disorders requiring hospital admission in Aragón between 2021 and 2024. Data from the Aragón Healthcare Big Data Platform (BIGAN) were analysed using descriptive statistics, multivariable logistic regression, and Poisson regression. **Results:** A total of 3057 incident cases were identified. The average annual incidence was 57.12 per 100,000 inhabitants in the overall adult population and 51.70 per 100,000 among adults aged ≥65 years. Older adults were more frequently women and showed higher rates of disability, polypharmacy, mood disorders (F3), and organic mental disorders (F0), whereas schizophrenia spectrum disorders (F2) and readmissions were less frequent. Organic mental disorders (OR = 12.28), mood disorders (OR = 2.50), disability (OR = 3.50), and polypharmacy (OR = 6.88) were independently associated with older age. Incidence remained relatively stable but showed geographical variation, with higher rates among older adults concentrated in predominantly mountainous counties. **Conclusions:** Adults aged ≥65 years showed an average annual incidence comparable to the overall adult population but exhibited a distinct clinical and geographical profile. These findings support integrated, age-sensitive, and territorially adapted mental healthcare strategies for ageing populations.

## 1. Introduction

Mental disorders (MDs) are characterized by clinically significant disturbances in cognition, emotional regulation, or behaviour that reflect dysfunction in the psychological, biological, or developmental processes underlying mental functioning and behaviour [1]. Mental disorders encompass a wide range of conditions, including anxiety disorders, personality disorders, obsessive–compulsive disorder, psychotic disorders, and schizophrenia [1,2].

Mental disorders represent one of the leading causes of disability worldwide and constitute a major public health challenge. According to the World Health Organization (WHO) [1], approximately 12.5% of the global population was affected by a mental disorder in 2019, including 301 million people with anxiety disorders, 280 million with depression, 40 million with bipolar disorder, and 24 million with schizophrenia [3]. Despite the availability of effective preventive and therapeutic interventions, many individuals do not have access to adequate mental healthcare. Furthermore, stigma, discrimination, and violations of human rights remain common among people living with mental disorders and are associated with poorer health outcomes, social exclusion, and increased suicide risk [1,3].

The burden of mental disorders is expected to increase in parallel with global population ageing [4]. Older adults frequently experience multimorbidity, frailty, cognitive decline, functional dependence, and social isolation, all of which may increase their vulnerability to mental health problems and complicate their management [5]. Mental disorders in later life are associated with reduced quality of life, increased healthcare utilization, institutionalization, and premature mortality [4,5]. Consequently, understanding the epidemiology of mental disorders has become increasingly important for planning age-sensitive healthcare services and mental health policies.

In Spain, mental and behavioural disorders affected approximately 34% of the population in 2023 [6]. Aragón, located in northeastern Spain, is one of the regions most affected by demographic ageing, characterized by a high ageing index, low population density in many rural areas, and marked territorial dispersion [7]. These demographic characteristics may contribute to increased vulnerability to mental disorders, particularly among older adults who often face barriers to accessing healthcare services and social support resources. Despite this context, information regarding the incidence and territorial distribution of mental disorders within Aragón remains scarce.

The occurrence and impact of mental disorders are influenced by multiple social and environmental determinants [8]. Previous studies have identified geographical differences in prevalence, with higher rates reported in Northern European countries and an increased risk of schizophrenia among individuals living in urban environments [9]. Socioeconomic inequalities also play an important role, as the prevalence of mental disorders increases with decreasing income levels and greater social vulnerability [2,9].

Individuals living with mental disorders experience substantial health and social disadvantages compared with the general population. They exhibit higher rates of premature mortality, unemployment, homelessness, and social exclusion [10]. For example, bipolar disorder is among the most common psychiatric conditions among people experiencing homelessness [11], while individuals diagnosed with schizophrenia show markedly lower employment rates than the general population [12]. These consequences may be particularly severe among older adults, who often face additional challenges related to physical comorbidities, dependency, and reduced social support networks.

Mental disorders also generate a considerable economic burden. In Spain, mental health problems account for more than EUR 45 billion annually, representing approximately 4.2% of the national gross domestic product [13]. Among hospitalised patients, the average length of stay has been estimated at 12.97 days, with a mean hospitalisation cost of EUR 9288.6 per patient [14]. Therefore, optimizing the organization and delivery of mental healthcare services has become a priority for healthcare systems.

Over recent decades, the transition from institutionalized care towards community-based mental health services has demonstrated positive outcomes, including reductions in hospital admissions, improvements in quality of life and treatment adherence, enhanced continuity of care, better access to services, and greater patient satisfaction [14]. Such approaches may be particularly beneficial for older adults, who frequently require coordinated, person-centred, and community-oriented care.

Given the progressive ageing of the Aragonese population and the increasing demand for mental healthcare services, understanding the epidemiological patterns and territorial distribution of mental disorders is essential for healthcare planning and resource allocation. Such evidence may contribute to identifying vulnerable populations, particularly older adults, and support the implementation of targeted interventions, community-based care models, and equitable healthcare policies.

Therefore, the aims of this study were to analyse the incidence of mental disorders in the Autonomous Community of Aragón (Spain), describe the sociodemographic and clinical characteristics of patients diagnosed and hospitalised with mental disorders, identify the diagnostic categories contributing to the burden of mental illness in the region, and compare temporal trends and geographical variations in incidence rates among adults aged ≥65 years.

## 2. Materials and Methods

### 2.1. Study Design and Population Characteristics

A retrospective, analytical, longitudinal observational study was conducted including adult patients (≥18 years) diagnosed with one of the mental disorders included in the predefined ICD-10-ES diagnostic categories (Table 1) and admitted to acute psychiatric inpatient units within the regional public healthcare network of Aragón (Spain), which provides specialized mental health services to approximately 1.3 million inhabitants, between 1 January 2021 and 31 December 2024. Patients with pre-existing cognitive impairment recorded as a clinical condition without an acute psychiatric admission and patients with a principal diagnosis of a substance use disorder, including disorders related to alcohol, opioids, cannabinoids, sedatives or hypnotics, cocaine and other stimulants, hallucinogens, and other psychoactive substances, were excluded from the study. However, patients whose principal diagnosis at hospital admission was classified as ICD-10-ES F0 (Organic Mental Disorders) were included, as these diagnoses constituted the incident mental disorder leading to hospitalisation. This distinction was made to avoid classifying pre-existing cognitive impairment without an acute psychiatric presentation as an incident mental disorder, while retaining organic mental disorders when they constituted the principal psychiatric diagnosis responsible for hospitalisation.

An incident case was defined as the first recorded diagnosis of a mental disorder (ICD-10-ES) requiring psychiatric hospitalisation between 1 January 2021 and 31 December 2024. To ensure that only incident cases were included, previous records available in the Aragón Healthcare Big Data Platform (BIGAN) were reviewed during the data extraction process, and patients with a mental disorder diagnosis recorded before the start of the study period were excluded. Subsequent hospital admissions occurring after the incident diagnosis during the study period were considered readmissions and were not counted as new incident cases.

The primary analysis included the entire adult study population (≥18 years). Adults aged ≥65 years constituted a predefined subgroup of this overall population and were therefore not an independent study cohort. A specific sub-analysis was performed in adults aged ≥65 years to further characterize their epidemiological and clinical profile and to evaluate temporal trends and geographical variations in the incidence of mental disorders across Aragón during the study period. Incidence was estimated separately for each calendar year using the annual number of incident cases, defined as first recorded diagnoses of mental disorders requiring hospital admission, and the corresponding annual population estimates.

### 2.2. Data Sources and Data Extraction

Following institutional authorization, data were obtained from the Aragón Healthcare Big Data Platform (BIGAN), a regional data warehouse that integrates anonymized information from the public healthcare system.

The extraction included clinical, sociodemographic, and healthcare-related variables recorded in hospital information systems. Diagnoses were coded according to the International Classification of Diseases, 10th Revision, Spanish Modification (ICD-10-ES) [15], in force during the study period.

Aggregated population data, including population density and territorial distribution by county, were obtained from the Geographical Institute of Aragón (IGEAR) through the Aragón Atlas and from the Aragón Statistics Institute [16]. Annual population estimates for the overall adult population and for adults aged ≥65 years were obtained from official regional demographic statistics and were used as denominators for the calculation of annual incidence rates.

### 2.3. Study Variables

The primary outcome variable was the principal mental disorder diagnosis at hospital admission, classified according to ICD-10-ES codes and grouped into diagnostic categories (Table 1). As independent variables, the following sociodemographic characteristics were collected: sex (female/male), age (years), county of residence (according to the official territorial division of Aragón), healthcare sector of residence (Teruel, Alcañiz, Huesca, Barbastro, Calatayud, Zaragoza I, Zaragoza II, and Zaragoza III), employment status (employed/pensioner), and annual income level based on pharmaceutical co-payment categories.

Clinical variables included psychiatric comorbidity, polypharmacy, disability status and degree of disability, institutionalization, and healthcare-related variables such as length of hospital stay and hospital readmissions during the study period. Psychiatric comorbidity was defined as the presence of one or more additional mental disorder diagnoses (ICD-10-ES F00–F99) recorded in addition to the principal diagnosis during the index hospital admission. Polypharmacy was defined as the concurrent use of five or more chronic medications, according to the Anatomical Therapeutic Chemical (ATC) Classification System [17], based on medication records available in the regional healthcare database.

In addition, contextual variables related to the residential environment were incorporated, including population density and number of inhabitants by county, obtained from official regional sources.

### 2.4. Ethical Considerations

The study was approved by the Research Ethics Committee of Aragón (Reference No. PI25/416). All procedures were conducted in accordance with the principles of the Declaration of Helsinki and applicable national data protection regulations. Confidentiality and data privacy were ensured in compliance with Organic Law 3/2018 on the Protection of Personal Data and Guarantee of Digital Rights. All data were anonymized prior to analysis, stored on secure password-protected servers with access restricted to the research team, and permanently deleted upon completion of the study.

### 2.5. Statistical Analysis

Statistical analyses were performed using Jamovi^®^ software (version 2.7.23). Descriptive analyses were conducted using absolute frequencies and percentages for categorical variables. Continuous variables were expressed as mean and standard deviation or median and interquartile range, depending on data distribution. Normality was assessed using the Shapiro–Wilk test.

For inferential analyses, associations between categorical variables were examined using the chi-square test or Fisher’s exact test, as appropriate. Comparisons of continuous variables were performed using Student’s *t*-test or one-way ANOVA for normally distributed data, and the Mann–Whitney U test or Kruskal–Wallis test when normality assumptions were not met. Pearson’s correlation coefficient (or Spearman’s rank correlation coefficient for non-normally distributed variables) was used to assess associations between continuous variables. Bonferroni correction was applied when multiple comparisons were performed.

Annual incidence rates were calculated for both the overall adult population and the predefined subgroup of adults aged ≥65 years as the number of incident cases of mental disorders per 100,000 inhabitants, using the corresponding annual population estimates as denominators. For descriptive purposes, the average annual incidence during the study period was calculated as the arithmetic mean of the annual incidence rates. No cumulative incidence over the four-year study period was calculated or compared between age groups.

To identify factors independently associated with older age, a multivariable binary logistic regression model was fitted with age group (≥65 years vs. <65 years) as the dependent variable. Sex, primary mental disorder diagnostic category (ICD-10-ES), hospital readmission, disability status, annual income level, and polypharmacy were entered simultaneously into the model as independent variables. Variables were selected based on their clinical relevance and availability in the study database. Adjusted odds ratios (ORs) and their corresponding 95% confidence intervals (95% CIs) were estimated. The <65-year age group was used as the reference category for the dependent variable, while the reference categories for categorical predictors were male sex, F2 (schizophrenia, schizoaffective disorders, and delusional disorders), no hospital readmission, no disability, annual income < EUR 18,000, and no polypharmacy. Model fit was assessed using deviance, Akaike’s Information Criterion (AIC), and McFadden’s pseudo-R^2^.

To evaluate temporal and territorial variations in annual incidence among adults aged ≥65 years, Poisson regression models were fitted using the number of incident cases as the dependent variable and the natural logarithm of the corresponding population aged ≥65 years as an offset term. Calendar year and county were included as explanatory variables. The Central county, which includes Zaragoza city and represents the most populous area in the region, was selected a priori as the reference category because its larger population and number of observed cases provided a stable reference for estimating relative incidence rates across counties. Thus, county-specific IRRs should be interpreted as relative incidence rates compared with Central rather than as direct urban–rural comparisons. Incidence rate ratios (IRRs) and their corresponding 95% confidence intervals (95% CIs) were estimated. Goodness-of-fit was evaluated using the residual deviance, the residual deviance-to-degrees-of-freedom ratio to assess potential overdispersion, and Akaike’s Information Criterion (AIC).

Missing data were handled using complete-case analysis. No imputation procedures were performed. For each analysis, only observations with complete information for the variables of interest were included; therefore, sample sizes may vary across analyses.

A two-sided *p*-value < 0.05 was considered statistically significant.

## 3. Results

### 3.1. Incidence of Mental Disorders in Aragón and General Characteristics of the Study Population

During the study period, a total of 3057 incident cases of mental disorders meeting the inclusion criteria were identified. The annual incidence was 64.49 per 100,000 inhabitants in 2021 (*n* = 859), 52.93 in 2022 (*n* = 703), 58.30 in 2023 (*n* = 782), and 52.75 in 2024 (*n* = 713), resulting in an annual average incidence of 57.12 during the study period.

Of all incident cases, 36.75% (*n* = 1123) occurred in men and 63.25% (*n* = 1933) in women. The mean age of the cohort was 48.66 ± 17.30 years. Women were significantly older than men (49.78 ± 17.14 vs. 46.77 ± 17.40 years; *p* < 0.001). The most represented age group was 45–65 years (40.53%), followed by 30–45 years (22.28%), >65 years (19.82%), and 18–30 years (17.37%).

Most cases were identified in urban areas (76.09%, *n* = 2240), whereas 23.91% (*n* = 704) were recorded in rural settings. Disability was uncommon, affecting only 3.70% (*n* = 113) of the study population.

The most frequent diagnostic categories were F3 (Mood Disorders; *n* = 1111) and F2 (Schizophrenia, Schizotypal and Delusional Disorders; *n* = 981). Significant sex differences were observed across diagnostic categories (*p* < 0.001). F2 disorders were proportionally more frequent among men (37.85%) than women (28.71%), whereas F3 disorders were more common among women (38.44%) than men (32.77%).

Age also differed significantly across diagnostic categories (*p* < 0.001). Organic Mental Disorders (F0) showed the highest mean age at diagnosis (67.08 ± 13.84 years), whereas Disorders of Psychological Development (F8) showed the lowest (29.79 ± 15.71 years).

Most patients did not present psychiatric comorbidity (62.15%, *n* = 1900), whereas 37.85% (*n* = 1157) had at least one additional mental health diagnosis. A significant association was observed between the primary diagnosis and the presence of psychiatric comorbidity (*p* < 0.001). Post hoc analysis revealed lower-than-expected rates of comorbidity among patients diagnosed with F2 disorders and higher-than-expected rates among those diagnosed with F4 (Neurotic, Stress-Related and Somatoform Disorders), F7 (Intellectual Disabilities), F9 (Behavioural and Emotional Disorders with Onset Usually Occurring in Childhood and Adolescence), and F99 (Unspecified Mental Disorder) after Bonferroni correction. These diagnostic categories contributed most strongly to the observed association.

No significant association was found between psychiatric comorbidity and polypharmacy (*p* = 0.284). The mean length of hospital stay was 18.16 ± 15.79 days. Most patients did not experience readmission (68.24%), while the remaining patients were readmitted during the study period.

Regarding occupational status, 52.17% (*n* = 1236) of patients were actively employed, whereas 47.83% (*n* = 1133) were retired or receiving a pension. From a socioeconomic perspective, most patients (71.06%, *n* = 1434) reported an annual income below EUR 18,000, while 28.34% (*n* = 572) reported incomes between EUR 18,000 and EUR 100,000. Only 0.59% (*n* = 12) reported annual incomes above EUR 100,000.

Most diagnoses were concentrated in the Central Aragón county, particularly for F0, F2, F3, F4, F5, F6, F8 and F9 disorders. Significant geographical variability was observed across counties and healthcare sectors (Table 2).

### 3.2. Geographical Distribution of Mental Disorders Across Aragón

Poisson regression analysis revealed marked geographical differences in mental disorder incidence across Aragón. The highest incidence rate ratios were observed in Sobrarbe (IRR = 2.89) and La Ribagorza (IRR = 2.78), while several counties showed incidence rates below that of the Central reference area (Table 3).

Temporal analyses showed that Sobrarbe consistently exhibited elevated incidence rates throughout the study period, while Campo de Borja, Valdejalón, and Bajo Aragón-Caspe consistently presented lower incidence rates.

Significant differences were also observed among healthcare sectors (*p* < 0.001). Schizophrenia spectrum disorders (F2) predominated in the Alcañiz, Calatayud, Zaragoza II, and Zaragoza III sectors, whereas Mood Disorders (F3) were the most prevalent diagnoses in Barbastro, Huesca, Teruel, and Zaragoza I.

### 3.3. Mental Disorders in Later Life: A Distinct Epidemiological Profile

Among adults aged 65 years and older, the average annual incidence of mental disorders during the study period was 51.70 per 100,000 inhabitants. Annual incidence rates remained relatively stable over time, with values of 49.53, 54.02, 52.72, and 50.89 cases per 100,000 inhabitants in 2021, 2022, 2023, and 2024, respectively. Although the highest annual incidence was observed in 2022, incidence remained broadly stable throughout the study period.

Of the 3057 incident cases included in the study, 606 (19.8%) occurred in adults aged 65 years or older, whereas 2451 (80.2%) were identified among individuals younger than 65 years. Older adults were more frequently women than men (68.6% vs. 61.9%, *p* = 0.002). No differences were observed between age groups regarding place of residence, with a similar distribution of urban and rural populations (*p* = 0.996).

Older adults showed a significantly higher prevalence of disability compared with younger individuals (7.8% vs. 2.7%, *p* < 0.001). In contrast, no significant differences were found in the mean number of psychiatric comorbidities between groups (1.12 ± 0.42 vs. 1.18 ± 0.58, *p* = 0.690).

The distribution of primary mental disorder diagnoses differed significantly according to age group (*p* < 0.001). Among older adults, mood disorders (F3) represented the most frequent diagnosis, accounting for 54.1% of cases, followed by schizophrenia, schizotypal and delusional disorders (F2; 20.5%) and neurotic, stress-related and somatoform disorders (F4; 11.6%). Organic mental disorders (F0) were markedly more common among older adults than among younger individuals (8.9% vs. 1.2%). Conversely, schizophrenia spectrum disorders (35.0% vs. 20.5%), personality and behavioural disorders in adults (7.0% vs. 1.2%), and neurotic, stress-related and somatoform disorders (16.3% vs. 11.6%) were proportionally more frequent among younger patients.

Polypharmacy was significantly more prevalent among older adults, affecting 96.0% of individuals aged 65 years or older compared with 90.4% of younger patients (*p* < 0.001). Although hospital readmission rates differed significantly between groups, the magnitude of this difference was small, with readmissions occurring in 28.4% of older adults and 32.6% of younger individuals (*p* = 0.046).

Marked differences were also observed in occupational and socioeconomic characteristics. As expected, most older adults were retired or receiving a pension (96.7%), whereas two-thirds of younger patients remained professionally active (66.8%) (*p* < 0.001). Regarding income level, older adults were less likely to report annual incomes below EUR 18,000 and more likely to belong to intermediate or high-income categories than younger individuals (*p* < 0.001).

No significant differences were found in the distribution of cases across healthcare sectors (*p* = 0.221), suggesting a relatively homogeneous territorial distribution of older adults with incident mental disorders throughout Aragón. Detailed comparisons between older and younger adults are presented in Table 4.

To identify factors independently associated with older age, a multivariable logistic regression analysis was performed including sex, primary mental disorder diagnosis, disability, polypharmacy, income level, and hospital readmission. The model demonstrated a moderate explanatory capacity (McFadden’s R^2^ = 0.138).

After adjustment, patients diagnosed with organic mental disorders (F0) were more than twelve times more likely to belong to the older adult group than those diagnosed with schizophrenia, schizotypal and delusional disorders (F2) (OR = 12.28, 95% CI: 6.81–22.15, *p* < 0.001). Likewise, mood disorders (F3) remained independently associated with older age (OR = 2.50, 95% CI: 1.80–3.47, *p* < 0.001). In contrast, personality and behavioural disorders in adults (F6) were significantly less frequent among older adults (OR = 0.18, 95% CI: 0.06–0.60, *p* = 0.005).

Disability (OR = 3.50, 95% CI: 1.88–6.50, *p* < 0.001) and polypharmacy (OR = 6.88, 95% CI: 1.65–28.68, *p* = 0.008) were independently associated with older age. Regarding socioeconomic status, individuals with annual incomes between EUR 18,000 and EUR 100,000 (OR = 1.83, 95% CI: 1.41–2.38, *p* < 0.001) and those with incomes above EUR 100,000 (OR = 4.92, 95% CI: 1.42–17.04, *p* = 0.012) showed higher odds of belonging to the older adult group compared with individuals earning less than EUR 18,000 per year.

Finally, hospital readmission was inversely associated with older age, with readmitted patients showing a lower likelihood of belonging to the older adult group (OR = 0.67, 95% CI: 0.51–0.89, *p* = 0.005). Sex was not independently associated with age group after adjustment for the remaining covariates (*p* = 0.423). The full results of the multivariable logistic regression analysis are presented in Table 5.

### 3.4. Geographical Distribution of Mental Disorder Incidence Among Older Adults

Poisson regression analysis adjusted for population size revealed no significant temporal changes in incidence rates between 2021 and 2024 (overall effect of year: χ^2^ = 0.499, *p* = 0.919). Compared with 2021, incidence rates were similar in 2022 (IRR = 1.07, 95% CI: 0.85–1.35), 2023 (IRR = 1.04, 95% CI: 0.83–1.30) and 2024 (IRR = 1.00, 95% CI: 0.79–1.26). In contrast, significant geographical variation was observed across countys (χ^2^ = 150.64, *p* < 0.001). The highest incidence rates were observed in Alto Gállego (IRR = 2.98, 95% CI: 1.92–4.63) and La Ribagorza (IRR = 3.66, 95% CI: 2.44–5.48), whereas lower rates were identified in Bajo Cinca, Cinco Villas, La Litera, Ribera Alta del Ebro and Valdejalón (Table 6).

## 4. Discussion

This study achieved its objectives by providing a comprehensive overview of the epidemiology of mental disorders in Aragón, describing the sociodemographic and clinical characteristics of affected patients, identifying the diagnostic categories contributing most to the burden of mental illness, and examining temporal and geographical variations in incidence among older adults. The findings demonstrate that mental disorders represent a substantial and sustained public health challenge across the region. The average annual incidence of mental disorders was 57.12 cases per 100,000 inhabitants in the general adult population and 51.70 cases per 100,000 among adults aged ≥65 years. Rather than indicating a higher incidence among older adults, our findings highlight that this population presents a distinct epidemiological and clinical profile, characterised by a higher proportion of mood disorders and organic mental disorders, greater disability, and increased polypharmacy. Although direct comparisons with previous studies should be interpreted cautiously because most have reported prevalence estimates rather than incidence rates and have primarily focused on specific conditions such as depression, anxiety, or dementia [18,19], population-based register studies conducted in other European settings provide relevant points of comparison. A nationwide Danish register study by Beck et al. examined age-specific incidence across a broad range of mental disorders and demonstrated substantial variation in incidence according to age, sex, and diagnostic category. Mood disorders were among the most frequent diagnostic groups, particularly among women [20]. Similarly, Stafford et al., in a population-based cohort of approximately three million people in Sweden, demonstrated that incident non-affective psychotic disorders can also occur in later life and identified important variation according to sex, age, and sociodemographic characteristics [21]. These findings support the importance of considering age- and diagnosis-specific epidemiological patterns when interpreting the burden of mental disorders in older populations. However, differences in case definitions and healthcare settings should be considered when comparing these European studies with our findings, as our analysis was specifically restricted to incident mental disorders requiring psychiatric hospitalisation.

Our findings are consistent with international evidence identifying older adults as a particularly vulnerable population and highlighting the growing impact of mental disorders in the context of population ageing [22]. Although incidence was not higher among older adults, mental disorders in later life remain associated with functional decline, loss of independence, increased healthcare utilisation, institutionalisation, and premature mortality, reinforcing the need for age-sensitive prevention and integrated models of care. These findings are particularly relevant in Aragón, one of the most rapidly ageing regions in Spain, where demographic ageing and territorial dispersion may further increase the demand for mental healthcare services.

Furthermore, annual incidence rates among older adults remained remarkably stable throughout the four-year study period, suggesting that the burden of mental disorders requiring psychiatric hospitalisation has remained persistent over time. This stability supports the implementation of age-specific prevention, early detection, and integrated care strategies in line with current international public health priorities [23].

The distinct epidemiological and clinical profile observed among older adults is likely explained by the combined influence of age-related neurobiological changes, the increasing prevalence of neurodegenerative diseases and multimorbidity, polypharmacy, and psychosocial factors such as social isolation and sensory impairment. Together, these factors increase vulnerability to mental disorders in later life and may contribute to the different diagnostic patterns identified in this age group [24,25].

Consistent with the distinct epidemiological profile observed in this study, women represented the majority of incident cases in both age groups, with an even greater predominance among adults aged ≥65 years. This finding is in line with previous epidemiological evidence showing that women experience a higher lifetime prevalence of affective disorders and are overrepresented in older age groups because of their longer life expectancy. Beyond these biological and demographic factors, gender-related social determinants may further increase the burden of mental disorders among older women. As discussed by Jin A. Lee et al. [26], widowhood, living alone, changes in family dynamics, and the progressive restructuring of social support networks contribute to increased psychological vulnerability through greater loneliness and social isolation. Their findings further suggest that these adaptations differ by sex: whereas proximity to children may mitigate loneliness among widowed men, older women rely more heavily on friendships and other voluntary social relationships, whose disruption has been associated with poorer mental health outcomes. These mechanisms may partly explain the greater proportion of women among older adults diagnosed with mental disorders and reinforce the importance of interventions aimed at strengthening social support networks and reducing loneliness as key components of healthy ageing and mental health promotion.

No significant differences were observed in the urban–rural distribution between younger and older adults, suggesting that the epidemiological profile of mental disorders among older adults in Aragón cannot be explained solely by place of residence. Current evidence indicates that mental health in later life is influenced less by rurality itself than by environmental and social contextual factors. In this regard, Ying et al. [27] reported that satisfaction with the community environment, including neighbourhood safety, green spaces, and residential conditions, was associated with a lower risk of depression among older adults. Similarly, Suh et al. [28] demonstrated that the availability of community healthcare, home-based services, social support resources, and adequate access to healthcare are key determinants of mental health in ageing populations. Functional decline and reduced mobility have also been identified as important contributors to psychological vulnerability in later life. Taken together, these observations indicate that the high incidence of mental disorders observed among older adults in Aragón is likely to reflect not only the effects of biological ageing but also the influence of environmental, functional, and healthcare-related factors, highlighting the need for comprehensive strategies that integrate medical care, community support, and healthy ageing initiatives.

Beyond the sociodemographic characteristics, older adults exhibited a distinct clinical profile compared with younger patients. The higher prevalence of disability and polypharmacy observed in this group is consistent with the greater burden of multimorbidity, frailty, and functional dependence associated with ageing. Previous studies have demonstrated a bidirectional relationship between frailty and depression, whereby frail older adults are more likely to develop depressive symptoms, while depression itself accelerates functional decline and disability [29,30]. This interaction suggests that physical and psychological vulnerability are closely interconnected in later life and may partly explain the greater complexity of mental disorders observed among older adults. Likewise, the higher prevalence of polypharmacy reflects the accumulation of chronic conditions and long-term pharmacological treatments. Recent evidence, including the study by Yu et al. [31], has consistently associated polypharmacy with an increased risk of cognitive impairment through adverse drug reactions, medication interactions, and prescribing cascades, which may have contributed to the marked increase in organic mental disorders (F0) identified in our study. These findings emphasise the importance of comprehensive geriatric assessment, medication review, and deprescribing strategies as part of routine mental healthcare for older adults.

Diagnostic patterns also differed substantially between age groups. Mood disorders (F3) were considerably more frequent among older adults, probably reflecting the cumulative impact of bereavement, loneliness, loss of autonomy, chronic physical illness, and progressive social role changes, all of which are recognised risk factors for late-life depression. This interpretation is supported by recent European evidence comparing older adults in Spain and Sweden [32], which identified loneliness as the strongest determinant of depressive symptoms regardless of the different sociocultural contexts of the two countries. Interestingly, despite a lower reported prevalence of loneliness than in Sweden, older adults in Spain exhibited a higher prevalence of depressive symptoms, suggesting that cultural, health-related, and social factors may modulate the relationship between loneliness and mental health. These findings reinforce the importance of addressing social isolation and psychosocial vulnerability as key targets for the prevention and management of mood disorders in later life [33]. In contrast, organic mental disorders (F0) showed the strongest association with older age, consistent with the increasing prevalence of cognitive impairment, neurodegenerative diseases, and acute confusional states in later life [34]. Dementia and delirium have a complex bidirectional relationship, with pre-existing cognitive impairment markedly increasing the risk of acute confusional episodes, which in turn may accelerate cognitive decline and functional deterioration [35]. These mechanisms may partly explain the markedly increased odds of F0 disorders observed in our study.

Conversely, schizophrenia and related disorders (F2) were less frequent among older adults, an expected finding given that the incidence of first-onset psychotic disorders declines markedly with advancing age. Interestingly, despite their greater clinical complexity, older adults experienced fewer hospital readmissions than younger patients (28.4% vs. 32.6%). Although this finding may appear counterintuitive, psychiatric readmission is influenced not only by overall clinical complexity but also by factors such as diagnostic profile, previous psychiatric admissions, symptom severity, disease trajectory, and post-discharge care. The lower readmission rate observed among older adults in our study may therefore partly reflect the different diagnostic distribution between age groups, particularly the lower proportion of schizophrenia and related disorders (F2), which are typically associated with recurrent psychiatric hospitalisation [36]. Differences in healthcare pathways after discharge, including greater use of community, geriatric, or long-term care services among older adults, may also contribute to this finding. However, these factors were not available in our database, and these potential explanations should therefore be interpreted cautiously and explored in future studies. Finally, older adults were more likely to belong to higher income categories despite being predominantly retired. Although lower socioeconomic position is a well-established determinant of poor mental health, our findings suggest that the relationship between income and mental disorders may differ in later life. Previous studies, such as that conducted by Sperandei et al. [37], have demonstrated that income is a major mediator of psychological distress in older adults and reflects not only current employment but also financial resources accumulated throughout the life course. In this context, the higher income categories observed in our older cohort may be explained by contributory pensions, accumulated savings, and property ownership rather than by a lower burden of mental illness. Consequently, our findings partially challenge the assumption that severe mental disorders are concentrated exclusively among the most socioeconomically disadvantaged groups and suggest that, after retirement, income should be interpreted within the broader context of lifetime socioeconomic accumulation rather than current occupational status. Beyond these individual-level characteristics, our findings also revealed important geographical differences in the distribution of mental disorders across Aragón, indicating that territorial context may represent an additional determinant of mental health in older adults.

The geographical analysis showed that the spatial distribution of incident mental disorders differed substantially between the overall adult population and older adults. In the overall population, several counties—including Sobrarbe, Ribagorza, Somontano, Alto Gállego, and Hoya de Huesca—showed higher incidence rate ratios than the Central reference area, whereas among adults aged ≥65 years the excess risk became concentrated almost exclusively in Alto Gállego and Ribagorza. Conversely, Sobrarbe, which exhibited the highest incidence in the overall population, was no longer significantly associated with increased incidence in older adults, suggesting that the geographical pattern observed in the overall population may differ according to age group. Likewise, the higher incidence previously observed in Somontano and Hoya de Huesca did not persist in the older population. Together, these findings indicate that the geographical pattern of mental disorder incidence changes with age, becoming concentrated in a small number of predominantly mountainous counties.

The persistence of significantly elevated incidence rates in Alto Gállego and Ribagorza across both age groups may reflect structural territorial factors operating alongside individual clinical characteristics. These two Pyrenean counties are characterised by pronounced population ageing, low population density, marked geographical dispersion, and greater distances to specialised healthcare services [38]. Given the unique demographic profile of Aragón, where several mountain areas combine advanced ageing with geographical isolation, these findings may have important implications for regional mental healthcare planning. Although the present study was not designed to identify the mechanisms underlying these territorial differences, previous evidence indicates that older adults living in rural and mountainous areas experience greater geographical isolation, reduced social support following rural depopulation, limited availability of specialised services, and increased barriers to accessing healthcare and community resources [39]. These contextual factors, together with frailty and functional decline, may increase psychological vulnerability and contribute to the higher incidence observed in northern Aragón. However, the observed geographical variation should be interpreted cautiously, as the ecological nature of this analysis does not allow causal relationships to be established. Differences in healthcare accessibility, availability of specialised mental health services, referral pathways, socioeconomic characteristics, healthcare-seeking behaviour, and local diagnostic or hospital admission practices may also have contributed to the variation observed between counties. Therefore, the higher incidence identified in some predominantly mountainous counties may reflect a combination of territorial, healthcare-related, socioeconomic, and population-level factors rather than a direct effect of geographical location itself. Strengthening community-based mental healthcare, improving coordination between health and social care services, and expanding accessible care models—including telemedicine—may therefore help reduce the territorial inequalities identified in this study [40].

The relative stability of annual incidence rates throughout the study period should also be considered in the context of the COVID-19 pandemic. Although the pandemic was associated with substantial changes in mental health needs and patterns of healthcare utilization, our findings did not show substantial temporal variation in the incidence of mental disorders requiring hospital admission between 2021 and 2024 [41]. This apparent stability may reflect the complex interaction between mental health needs and pandemic-related changes in access to and utilization of psychiatric inpatient services. However, because the study period began in 2021 and did not include a pre-pandemic reference period, our data do not allow us to determine whether the pandemic itself affected the underlying incidence of severe mental disorders requiring hospitalisation. Further studies incorporating pre-pandemic data are needed to specifically assess the impact of COVID-19 on these temporal patterns.

This study has several strengths. To our knowledge, it is one of the first population-based studies to comprehensively compare the epidemiological, clinical, and geographical patterns of incident mental disorders between the overall adult population and adults aged ≥65 years within the same healthcare system. The inclusion of all incident cases requiring hospital admission across Aragón over a four-year period, together with the use of routinely collected regional healthcare data, provided robust population-based estimates and enabled the simultaneous evaluation of temporal trends, territorial variability, and age-related differences in a large real-world cohort. However, several limitations should be acknowledged. First, the retrospective observational design precludes establishing causal relationships. Second, the use of administrative healthcare databases may be subject to coding inaccuracies, diagnostic misclassification, variability in routine clinical coding, and incomplete recording of relevant clinical variables. In addition, direct measures of psychiatric symptom severity were not available in the database, limiting our ability to account for differences in clinical severity between patients. This limitation may be particularly relevant to the classification of organic mental disorders (F0), as this category encompasses heterogeneous clinical conditions and relies on diagnostic coding recorded in routine clinical practice. Although the inclusion criteria required F0 to be recorded as the principal diagnosis leading to psychiatric hospitalisation, some degree of diagnostic coding variability or misclassification within this category cannot be excluded. Therefore, findings related to F0 disorders should be interpreted with appropriate caution. Furthermore, the exclusion of patients with pre-existing cognitive impairment without an acute psychiatric admission may have influenced the epidemiological profile observed among older adults, in whom cognitive impairment is particularly prevalent. However, this criterion was necessary to distinguish pre-existing cognitive conditions from incident mental disorders requiring psychiatric hospitalisation. Additionally, the multivariable logistic regression model showed a moderate explanatory capacity (McFadden’s pseudo-R^2^ = 0.138), suggesting that, although several variables included in the model were independently associated with older age, additional biological, psychosocial, environmental, and healthcare-related factors not available in the present database are likely to contribute to the observed differences between age groups. Finally, restricting the study population to incident cases requiring hospital admission may have introduced selection bias by preferentially capturing patients with more severe or acute mental disorders, while excluding individuals managed exclusively in outpatient or community-based settings. Consequently, the findings primarily reflect the epidemiological and clinical characteristics of mental disorders requiring inpatient psychiatric care and may not be generalizable to the full spectrum of mental disorders in the general population. Overall, our findings indicate that the burden of mental disorders in older adults arises from the complex interaction of biological ageing, clinical complexity, social vulnerability, and territorial inequalities. From a clinical perspective, these findings support the incorporation of age-sensitive assessment into routine mental healthcare for older adults, with particular attention to mood and organic mental disorders, disability, multimorbidity, and polypharmacy. Comprehensive geriatric assessment, regular medication review, early identification of functional and cognitive decline, and coordination between psychiatric, primary care, geriatric, and community services may help address the greater clinical complexity observed in this population. These results reinforce the need for integrated, age-sensitive, and territorially adapted mental healthcare strategies capable of addressing the growing demands of an ageing population while reducing geographical and social disparities in access to care. Despite these contributions, several questions regarding the mechanisms underlying the observed territorial differences remain unanswered. Future research should include prospective studies to identify the individual-, healthcare-, and contextual-level determinants underlying the geographical variation observed in this study. In particular, the role of healthcare accessibility, service availability, referral pathways, socioeconomic conditions, social support, and clinical severity should be investigated to determine whether these factors contribute to regional differences in the incidence of mental disorders requiring hospitalisation.

## 5. Conclusions

This population-based study provides one of the first comprehensive comparisons of the epidemiological, clinical, and geographical patterns of incident mental disorders between the overall adult population and adults aged ≥65 years within the same healthcare system. Although annual incidence rates were similar between age groups and remained stable throughout the study period, older adults exhibited a distinct epidemiological and clinical profile characterised by a higher prevalence of mood and organic mental disorders, greater disability, and increased polypharmacy. Geographical analyses further revealed that, among older adults, higher incidence rates were concentrated in a small number of predominantly mountainous counties, suggesting that territorial context may contribute to mental health inequalities beyond individual clinical factors.

These findings highlight the need for integrated, age-sensitive, and territorially adapted mental healthcare strategies that combine prevention, early detection, comprehensive geriatric assessment, and community-based services. Such approaches may help address the growing complexity of mental disorders in ageing populations while reducing geographical inequalities in access to mental healthcare.

## Figures and Tables

**Table 1:** ICD-10-ES codes.

F0	organic mental disorders
F2	schizophrenia, schizoaffective disorders, delusional disorders
F3	mood disorders
F4	neurotic, stress-related, and somatoform disorders
F5	behavioral disorders associated with physiological dysfunctions and somatic factors
F6	personality and behavioral disorders in adults
F7	intellectual disability
F8	psychological developmental disorders
F9	behavioral and emotional disorders that begin in childhood and adolescence
F99	unspecified mental disorder

**Table 2:** Sociodemographic, Clinical, and Geographic Characteristics of Incident Mental Disorder Cases in Aragón (2021–2024).

Variable		
Sex N (%)	Male	1123 (36.75)
	Female	1933 (63.25)
Area of residence N (%)	Rural	704 (23.91)
	Urban	2240 (76.09)
Disability N (%)	Yes	113 (3.70)
	No	2944 (96.30)
Primary mental disorder diagnosis N (%)	F0	84 (2.75)
	F2	981 (32.09)
	F3	1111 (36.34)
	F4	469 (15.34)
	F5	5 (0.16)
	F6	179 (5.86)
	F7	45 (1.47)
	F8	34 (1.11)
	F9	75 (2.45)
	F99	29 (0.95)
Comorbidities	Mean (SD)	1.17 ± 0.56
Polypharmacy N (%)	Yes	2798 (91.53)
	No	259 (8.47)
Hospital readmission N (%)	Yes	971 (31.76)
	No	2086 (68.24)
Employment status N (%)	Employed	1236 (52.17)
	Pensioner	1133 (47.83)
Income level N (%)	<EUR 18,000	1434 (71.06)
	EUR 18,000 and EUR 100,000	572 (28.34)
	EUR 100,000	12 (0.59)
Healthcare Sector N (%)	Alcañiz	76 (2.85)
	Barbastro	357 (12.12)
	Calatayud	57 (1.93)
	Huesca	314 (10.66)
	Teruel	198 (6.72)
	Zaragoza 1	557 (18.91)
	Zaragoza 2	843 (28.62)
	Zaragoza 3	544 (18.47)

Note: The total study population comprised 3057 incident cases of mental disorders recorded in Aragón between 2021 and 2024. The analyses presented in this table were performed using the available data for each variable. A small number of cases were excluded because of missing or incomplete information; therefore, the denominators may differ from the total study population. SD, standard deviation; F0, Organic, including symptomatic, mental disorders; F2, Schizophrenia, schizotypal and delusional disorders; F3, Mood (affective) disorders; F4, Neurotic, stress-related and somatoform disorders; F5, Behavioural syndromes associated with physiological disturbances and physical factors; F6, Disorders of adult personality and behaviour; F7, Intellectual disabilities; F8, Disorders of psychological development; F9, Behavioural and emotional disorders with onset usually occurring in childhood and adolescence; F99, Unspecified mental disorder.

**Table 3:** County-specific incidence cases, incidence rate ratios (IRRs), and heat map of mental disorders in Aragón.

Region	Incident Cases (N)	IRR
Alto Gállego	45	1.39
Andorra-Sierra de Arcos	11	0.64
Aranda	6	0.77
Bajo Aragón	34	0.51
Bajo Aragón-Caspe	16	0.45
Bajo Cinca	28	0.48
Bajo Martín	3	0.40
Campo de Belchite	11	1.04
Campo de Borja	4	0.24
Campo de Cariñena	19	0.81
Campo de Daroca	4	0.62
Central	1770	Reference
Cinca Medio	56	0.99
Cinco Villas	47	0.66
Comunidad de Calatayud	45	0.53
Comunidad de Teruel	155	1.45
Cuencas Mineras	11	0.79
Gúdar-Javalambre	13	0.73
Hoya de Huesca	212	1.32
Jacetania	41	0.96
Jiloca	14	0.48
La Litera	44	1.01
La Ribagorza	81	2.78
Maestrazgo	4	0.77
Matarraña	8	0.41
Monegros	19	0.44
Ribera Alta del Ebro	35	0.54
Ribera Baja del Ebro	7	0.66
Sierra de Albarracín	5	0.48
Sobrarbe	52	2.89
Somontano de Barbastro	97	1.74
Tarazona y el Moncayo	17	0.52
Valdejalón	30	0.43

Note: The study included 3057 incident cases of mental disorders recorded in Aragón between 2021 and 2024. The geographical analysis by county was based on the 2944 cases with available county-of-residence information. A total of 113 cases (3.7%) were excluded from this analysis because geographical information was missing or could not be assigned to a specific county. IRR, incidence rate ratio. Central, the most populous area in Aragón, was used as the reference category (IRR = 1.00). County-specific IRRs represent incidence rates relative to Central and should not be interpreted as direct urban–rural comparisons. Cell colours indicate the magnitude of the IRR: green, IRR < 1.00; yellow, IRR 1.00–1.99; red, IRR ≥ 2.00.

**Table 4:** Sociodemographic, clinical, and healthcare characteristics of patients with incident mental disorders according to age group (<65 years vs. ≥65 years).

Variable		<65 Years (*n* = 2451; 80.2%)	≥65 Years (*n* = 606; 19.8%)	*p*
Sex *n* (%)	Male	933 (38.10)	190 (31.40)	0.002
	Female	1517 (61.90)	416 (68.6)	
Area of residence *n* (%)	Rural	562 (23.90)	142 (23.90)	0.996
	Urban	1788 (76.10)	452 (76.10)	
Disability *n* (%)	Yes	66 (2.70)	47 (7.80)	<0.001
	No	2385 (97.30)	559 (92.20)	
Primary mental disorder diagnosis *n* (%)	F0	30 (1.20)	54 (8.90)	<0.001
	F2	857 (35.0)	124 (20.50)	
	F3	783 (31.90)	328 (54.10)	
	F4	399 (16.30)	70 (11.60)	
	F5	49 (2)	1 (0.20)	
	F6	172 (7)	7 (1.20)	
	F7	44 (1.80)	1 (0.20)	
	F8	31 (1.30)	3 (0.50)	
	F9	64 (2.60)	11 (1.80)	
	F99	22 (0.90)	7 (1.20)	
Comorbidities	Mean (SD)	1.18 ± 0.58	1.12 ± 0.42	0.690
Polypharmacy *n* (%)	Yes	2216 (90.40)	582 (96)	<0.001
	No	235 (9.60)	24 (4)	
Hospital readmission *n* (%)	Yes	799 (32.60)	172 (28.40)	0.046
	No	1652 (67.40)	434 (71.60)	
Employment status *n* (%)	Employed	1218 (66.80)	18 (3.30)	<0.001
	Pensioner	606 (33.20)	527 (96.70)	
Income level *n* (%)	<EUR 18,000	1241 (74.30)	193 (55.60)	<0.001
	EUR 18,000 and EUR 100,000	423 (25.30)	149 (42.90)	
	EUR 100,000	7 (0.40)	5 (1.40)	
Healthcare Sector *n* (%)	Alcañiz	54 (2.30)	22 (3.70)	0.221
	Barbastro	298 (12.70)	59 (9.90)	
	Calatayud	41 (1.70)	16 (2.70)	
	Huesca	250 (10.60)	64 (10.80)	
	Teruel	161 (6.80)	37 (6.20)	
	Zaragoza 1	448 (19)	109 (18.40)	
	Zaragoza 2	670 (28.5)	173 (29.10)	
	Zaragoza 3	430 (18.3)	114 (19.20)	

**Abbreviations:** SD, standard deviation; F0, Organic Mental Disorders; F2, Schizophrenia, Schizotypal and Delusional Disorders; F3, Mood Disorders; F4, Neurotic, Stress-Related and Somatoform Disorders; F5, Behavioural Syndromes Associated with Physiological Disturbances and Physical Factors; F6, Personality and Behavioural Disorders in Adults; F7, Intellectual Disabilities; F8, Disorders of Psychological Development; F9, Behavioural and Emotional Disorders with Onset Usually Occurring in Childhood and Adolescence; F99, Unspecified Mental Disorder. **Note:** Values are expressed as *n* (%) or mean ± SD. Differences between age groups (<65 years and ≥65 years) were assessed using Pearson’s χ^2^ test or the Mann–Whitney U test, as appropriate. *p*-values < 0.05 were considered statistically significant.

**Table 5:** Factors independently associated with older age (≥65 years): multivariable logistic regression analysis.

Variable	Adjusted OR	95% CI	*p*-Value
Disability (yes)	3.50	1.88–6.50	<0.001
Polypharmacy (yes)	6.88	1.65–28.68	0.008
Readmission (yes)	0.67	0.51–0.89	0.005
Income EUR 18,000–EUR 100,000	1.83	1.41–2.38	<0.001
Income > EUR 100,000	4.92	1.42–17.04	0.012
F0 vs. F2	12.28	6.81–22.15	<0.001
F3 vs. F2	2.50	1.80–3.47	<0.001
F6 vs. F2	0.18	0.06–0.60	0.005

**Abbreviations:** OR, odds ratio; CI, confidence interval. **Reference categories:** schizophrenia, schizotypal and delusional disorders (F2), annual income < EUR 18,000, absence of disability, absence of polypharmacy, and no hospital readmission. **Model performance:** McFadden’s pseudo-R^2^ = 0.138.

**Table 6:** Poisson regression analysis of territorial variations in mental disorder incidence among adults aged ≥65 years in Aragón *.

County	IRR	95% CI	*p*-Value
Central (reference)	1.00	Reference	—
Alto Gállego	2.98	1.92–4.63	**<0.001**
Andorra-Sierra de Arcos	0.25	0.03–1.76	0.162
Aranda	0.50	0.07–3.53	0.483
Bajo Aragón	0.55	0.27–1.11	0.094
Bajo Aragón-Caspe	0.65	0.27–1.58	0.343
Bajo Cinca	0.25	0.08–0.79	**0.018**
Bajo Martín	0.00 *	—	0.994
Campo de Belchite	0.00 *	—	0.992
Campo de Borja	0.26	0.04–1.83	0.176
Campo de Cariñena	0.73	0.27–1.96	0.535
Campo de Daroca	0.51	0.07–3.66	0.506
Cinca Medio	1.27	0.76–2.13	0.364
Cinco Villas	0.48	0.24–0.96	0.037
Comunidad de Calatayud	0.69	0.41–1.16	0.158
Comunidad de Teruel	1.17	0.80–1.72	0.422
Cuencas Mineras	1.13	0.42–3.02	0.815
Gúdar-Javalambre	0.75	0.24–2.32	0.612
Hoya de Huesca	0.83	0.57–1.21	0.338
Jacetania	0.87	0.43–1.76	0.700
Jiloca	0.26	0.06–1.03	0.056
La Litera	0.10	0.01–0.68	**0.019**
La Ribagorza	3.66	2.44–5.48	**<0.001**
Maestrazgo	2.53	0.94–6.77	0.066
Matarraña	0.74	0.28–1.99	0.553
Monegros	0.49	0.22–1.11	0.087
Ribera Alta del Ebro	0.23	0.07–0.72	0.012
Ribera Baja del Ebro	0.78	0.20–3.15	0.732
Sierra de Albarracín	0.00 *	—	0.992
Sobrarbe	1.91	0.95–3.84	0.071
Somontano de Barbastro	0.60	0.30–1.21	0.152
Tarazona y el Moncayo	0.38	0.12–1.18	0.095
Valdejalón	0.16	0.04–0.65	**0.010**

* Estimates not interpretable due to sparse data and unstable model estimates. **Note:** Bold values indicate statistical significance (*p* < 0.05).

## Data Availability

The data presented in this study are not publicly available because they contain anonymized healthcare information obtained from the Aragón Healthcare Big Data Platform (BIGAN) under institutional authorization. Access to these data is subject to the policies and approval procedures of the Aragón Health Service (Servicio Aragonés de Salud).

## Abbreviations

The following abbreviations are used in this manuscript: ATC: Anatomical Therapeutic Chemical; BIGAN: Aragón Healthcare Big Data Platform; CI: Confidence Interval; ICD-10-ES: International Classification of Diseases, 10th Revision, Spanish Clinical Modification; IGEAR: Geographical Institute of Aragón; IRR: Incidence Rate Ratio; MDs: Mental Disorders; OR: Odds Ratio; SD: Standard Deviation; WHO: World Health Organization.
